# Introducing Voluntary Assisted Dying: Staff Perspectives in an Acute Hospital

**DOI:** 10.34172/ijhpm.2020.216

**Published:** 2020-11-22

**Authors:** Robin Digby, Rosalind McDougall, Michelle Gold, Danielle Ko, Lisa O’Driscoll, Tracey Bucknall

**Affiliations:** ^1^School of Nursing and Midwifery, Deakin University, Geelong, VIC, Australia.; ^2^Centre for Quality and Patient Safety Research (QPS), Alfred Health Partnership, Melbourne, VIC, Australia.; ^3^Melbourne School of Population and Global Health, University of Melbourne, Melbourne, VIC, Australia.; ^4^Alfred Health Partnership, Melbourne, VIC, Australia.; ^5^Austin Health, Melbourne, VIC, Australia.; ^6^Department of Palliative Care, Austin Health, Melbourne, VIC, Australia.; ^7^School of Nursing and Midwifery, Faculty of Health, Deakin University, Geelong, VIC, Australia.

**Keywords:** Euthanasia, Clinician Perspective, Acute Hospital, Clinical Decision-Making, End-of-Life, Assisted Suicide

## Abstract

**Background:** Voluntary assisted dying (VAD) was legalised in Victoria, Australia in June 2019. Physicians can now assist patients to end their lives by providing drugs for self-administration at their voluntary and competent request (or for physician administration in limited circumstances). This study investigates the opinions of clinicians on the implementation of the legislation in one Victorian hospital.

**Methods:** This exploratory survey study was conducted at a 600-bed acute hospital in Melbourne, Australia in Jan 2019. 382 clinicians completed one or more qualitative questions. Participants commented on VAD, potential workplace challenges and staff support required. Free-text responses were analysed using inductive content analysis.

**Results:** Six themes: (1) Polarised views; (2) Fear of conflict; (3) Emotional burden; (4) Vulnerable patients; (5) Organisational challenges; (6) Decision-making. There were diverse views including objections to VAD for religious or ethical reasons, and whole-hearted support based on a compassionate response to suffering and the right of patients to self-determination. Participants feared conflict between colleagues, families and patients, and aggression towards staff. Clinicians called for educational and psychological support. There was concern that vulnerable patients may be coerced to opt for VAD to lessen the burden on families or the health system. Clinicians feared workloads would increase with the introduction of VAD. Patient decision-making capacity in this context must be firmly established before proceeding, and thorough assessments for depression, and optimal symptom management must be implemented before VAD is approved. A dedicated VAD team was suggested to support staff and manage VAD patients.

**Conclusion:** Participants expressed polarised opinions about VAD and showed considerable anxiety about its introduction. Additional education and support are required to ensure that clinicians understand details of the legislation and their professional and personal options. Tolerance and respect for alternative viewpoints must be advocated within the organisation and more broadly.

## Background

**Key Messages**

** Implications for policy makers**

- Voluntary assisted dying (VAD) is a divisive issue which requires sensitive handling to accommodate alternative views and ensure that respect and tolerance are maintained.
- There was a united view that patients facing unbearable suffering at the end of life should have their needs met and suffering reduced.
- Guidelines for determining and assessing patient capacity for decision-making in this context are sought, to protect patients from coercion and clinicians from potential repercussions.
- Clinicians require considerable psychological and educational support when involved with VAD.

** Implications for the public**

Voluntary assisted dying (VAD) is available to Victorian adults who are experiencing unbearable suffering in the final 6 months of life and have the capacity to make the decision to take this option. However, support for VAD is far from universal among clinicians and the community, making tolerance and respect for diverse views essential.

Voluntary assisted dying (VAD) became legally available in the state of Victoria, Australia in June 2019 after the law was passed in late 2017. Legalising VAD is becoming increasingly common in jurisdictions around the world including Switzerland, the Netherlands, Belgium, Luxembourg, Colombia, Canada and ten states in the United States where it has been sanctioned in various forms, in some cases for many years.^1^ Victoria was the first Australian state to approve VAD since legislation was approved in the Northern Territory in 1997 and then overturned not long after being introduced.^2^ The *Voluntary Assisted Dying Act 2017 (Victoria)* was passed in November 2017, with an 18-month window for planning implementation. From June 19, 2019, Victorians who are at the end of life and comply with strict criteria, can request physicians to prescribe a lethal dose of medication for them to ingest, or in limited circumstances, request physician assistance to administer a lethal substance.^3^

 In Victoria, VAD means ‘the administration of a VAD substance and includes steps reasonably related to such administration.’^4^ The Victorian Act allows adult Australian citizens or permanent residents, currently resident in Victoria, having decision-making capacity, in the final weeks or months of life, with a serious and incurable condition causing enduring and unbearable suffering that cannot otherwise be adequately relieved to choose the option of VAD.^1^ In limited circumstances this may include the assistance of a medical practitioner to administer the substance, however in most cases the patient will self-administer. Mental illness alone is not an eligible condition. The Act permits healthcare practitioners who object to VAD to decline participation in any or all of the VAD processes.^2^

 There is an assessment process for people seeking VAD which includes evaluation by two medical specialists, one of whom is the coordinating practitioner overseeing the process, and the other the consulting practitioner who provides an opinion on whether the patient meets the eligibility criteria.^2^ In the event that the patient is unable to physically self-administer or digest the substance, the coordinating practitioner may administer it to them. The patient must make three formal requests for VAD, two verbal and one written, before VAD is approved. If the patient is deemed to be eligible, the coordinating practitioner must then apply for a permit. A pharmacist will dispense and counsel the patient about the drug to be used.

 There has been an increasing acceptance of VAD in Australia as community attitudes change and the right to personal choice is more widely recognised.^3^ However, despite the VAD regulations in Victoria being very conservative compared to other jurisdictions around the world local support for VAD is not universal. There continues to be robust debate and wide-ranging views and it is recognised that the introduction of VAD presents a substantial shift in policy and practice in Victorian hospitals. Significant support is required for staff navigating the practical implications, including respect for differing moral and ethical positions.

 At the time of writing, Victoria is the only Australian state in which VAD is available. Western Australia recently passed VAD legislation with an intended start date of mid-2021.^4^ Consequently there is limited local data available to inform hospital approaches to implementation despite the shared goal of reducing suffering and providing good dying experiences for patients.^3,5,6^ The experiences of clinicians in other jurisdictions have been mixed, with some experiencing considerable difficulties in working relationships,^7-9^ whilst others found that relationships with colleagues could also be strengthened^7^. For some there was a lack of clarity in their role and scope of practice.^10^ Many clinicians experienced complex emotions^9^ including both profound feelings of professional fulfilment and emotional distress.^8^

 Professional bodies differ in their attitudes towards VAD. Objections and concerns have been outlined in the position statements of a number of groups including the Australian Medical Association which takes the position that doctors should not be involved in interventions that have the ending of a person’s life as their primary intention.^11^ The International Association for Hospice and Palliative Care raised concerns that the inadequacy of palliative services should be addressed before VAD is considered.^12^ However, Palliative Care Australia^13^ was equivocal, declaring neither support nor opposition to VAD but emphasised tolerance for the views of others. The Australia and New Zealand Society for Geriatric Medicine expressed concern about the risk to vulnerable patients, especially the challenges estimating prognosis, and capacity to make an informed decision.^14^

 As the implementation of the VAD legislation is in its nascency in Australia, exploring the opinions of clinical staff, and identifying and addressing their concerns are important steps in ensuring VAD is appropriately delivered to those who have the legal right to receive it.

## Methods

 The objective of this research is to explore the opinions of clinical staff on the implementation of the new VAD legislation in one Victorian healthcare institution in order to contribute information to ensure the ethical implementation of VAD according to the Victorian law. The study was undertaken during the period between the passing of the law in late 2017 and the start date in 2019 when organisations were making decisions about participating in VAD.

###  Research Question 

 This study investigates the following research question: What are the views of clinical staff about the potential impact of new Victorian legislation allowing VAD?

 The study aims to:

1. Capture the views of clinical staff about the implementation of VAD in their hospital.
2. Identify perceived challenges that this legislative change will create for clinical staff in this institution.
3. Seek input from clinicians about local staff support required in the context of this change.
4. Inform the hospital’s approach to implementing VAD legislation and contribute to evidence about this issue more broadly.

###  Design and Study Methodology

 This study is part of a broader mixed methods survey project, conducted in seven health services in Victoria, Australia, and reports the qualitative data from one institution. Other qualitative and quantitative results from the project are reported elsewhere.^15^ The addition of detailed and personalised qualitative or contextualised accounts to survey data strengthens the research^16^ and is useful when investigating a complex issue.^17^ This study used inductive content analysis to examine the free-text responses of staff to the survey in one health service to gain greater understanding of staff views across disciplines in a single institutional context.

###  Study Setting

 This article examines the views of clinical staff at one 600 bed, major metropolitanMelbourne hospital which provides a comprehensive range of specialist acute health and mental health services with approximately 100 000 admissions per year.

###  Participants

 The participants were clinical staff including medical, nursing, allied health, pastoral care, and pharmacy. The participants were recruited using two strategies:

1. An email was sent to all clinical staff from the Chief Medical Officer, Chief Nurse, Director of Allied Health and Director of Pharmacy with at least one reminder follow-up email. The survey was open January 2019.
2. A link to the survey was available on the staff intranet for the three-week period.

###  Inclusion and Exclusion Criteria

 All clinical staff currently working at the hospital met the inclusion criteria. Staff without a clinical role were excluded.

###  Sampling and Data Source

 This was a voluntary survey, to which a total of 1229 clinicians responded at this hospital. The survey contained six demographic questions followed by multichoice questions related to the legislation and the participant’s view on potential involvement in VAD, plus four qualitative questions with free text responses (Box 1). Response to the free text questions was not forced by the logic of the survey. More than half the participants (56%, n = 382) responded to one or more qualitative questions, forming the sample for this study.

**Box 1. Free Text Survey Questions**

- Overall, what is your position on Victoria’s recent legalisation of VAD?
- What challenges (if any) do you envisage VAD will create in your work?
- What support should be developed to help staff deal with the challenges you have identified?
- Do you have any other comments in relation to the issues raised in this survey?

Abbreviation: VAD, voluntary assisted dying.

###  Data Analysis and Interpretation

 The qualitative data obtained from the free-text section of the surveys were analysed using inductive content analysis.^18^ This systematic and flexible approach avoids pre-conceived themes but allows the themes to become apparent as the researcher becomes immersed in the data and new insights become evident.^19^

 To commence the process, one author (RD) read and re-read the free-text responses to the survey questions to gain an overall sense of the data. A comprehensive table of selected excerpts was sent to the other authors who read and made notes of potential themes. Discussions within the research group lead to further refinements until themes were clarified and there was consensus. Initially a large number of potential themes were identified, but on further examination of the data, they were combined, refined and subthemes identified.

 Trustworthiness and rigour were demonstrated through several strategies including peer review of the data coding, use of an audit trail and demonstration of authenticity by fairly and faithfully describing a range of realities.^20^ The authors discussed the data analysis, comparing, questioning and defending the analysis until consensus was reached and the final themes were agreed.^20^

## Results

 Clinical staff from a wide range of disciplines including medical interns, registrars and consultants, nurses, and allied health staff (social workers, physiotherapists, occupational therapists, dietitians, speech pathologists, neuropsychologists, psychologists, pharmacists and radiation therapists) (n = 382) provided qualitative data (Table 1).

**Table 1:** Demographics and Disciplines of Staff Members (N = 382)

**Age (y)**	**No. (%)**
30 or below	131 (34.29)
31-40	108 (28.27)
41-50	77 (20.16)
51-60	46 (12.04)
60+	20 (5.24)
**Role **	
Nurse	217 (56.81)
Allied health	86 (22.51)
Medical specialist	50 (13.09)
Junior doctor - fellow, registrar (advanced trainee)	10 (2.62)
Junior doctor - intern, resident, registrar (basic trainee)	19 (4.97)

 The responses reflected a significant amount of fear and uncertainty associated with this legislation. Many respondents had limited understanding of VAD and the potential ramifications for clinical practice, and it was evident that there were a number of misunderstandings of the content of the legislation. Some respondents had very strong views either against or in favour of VAD, however many were unsure. The data were categorised into six main themes with several sub-themes underpinning them: (1) Fear of conflict; (2) Polarised views; (3) Emotional burden; (4) Vulnerable patients; (5) Organisational challenges; (6) Decision-making (Table 2).

**Table 2:** Themes and Subthemes

**Fear of Conflict**	**Polarised Views**	**Emotional Burden**	**Vulnerable Patients**	**Organisational Challenges**	**Decision-Making**
Between staff Between/ with families In the community With patients who don’t qualify for VAD	Ethical, moral and religious objections Transgressing the Hippocratic Oath, patient trust Strong supporters of VAD	Support for staff Support for patients and families	Pressure from family to undergo VAD Concern that other options may not have been tried	Physical resources Staff education Capacity/ resources -specialist VAD team Storage and management of the drug	Patient capacity often unclear Patient prognosis often unclear Uncertainty

Abbreviation: VAD, voluntary assisted dying.

###  Fear of Conflict 

 Many respondents believed that the different views of staff about this issue would cause conflict in the workplace by creating a group who refused to care for VAD patients and others who were consequently obliged to take on more of this work. Staff with a religious or moral objection were fearful that this tension would impact on their work environment and may lead to bullying. Those who were in favour of the legislation similarly feared recriminations from colleagues.

*“It may cause heated debate among those pro and opposed to the practice. This could lead to conflict that could be potentially very difficult to resolve as it involves peoples’ closely held personal, and sometimes religious, beliefs” *(Nurse).

 One staff member mentioned that they would be forced to declare their personal views which they preferred to keep private.

*“I prefer to keep my thoughts, beliefs and opinions to myself” *(Allied Health – Pastoral Care).

 The opinions of colleagues were important to some staff who felt that they might be ostracised when their views were made known.

*“Another challenge could be fighting against the feeling of being an outsider because of not supporting this legislation and feeling like others see me as being dogmatic and unsympathetic” *(Junior Medical Officer).

 There were staff members who were supportive of VAD in theory but preferred not to be involved in a hands-on way. If a large number of staff had religious and ethical objections, the workload of those remaining could potentially be impacted.

*“I don’t doubt that there will be many staff who might object to participating in VAD, based on their own cultural or spiritual beliefs… I believe this will put added pressure on other staff members who are unsure of their stance on VAD…I believe this could negatively impact the emotional wellbeing of staff” *(Nurse).

 There was concern about family conflict from a number of standpoints. There could be disagreement between family members, or between family members and the patient.

*“I imagine family members would be challenging in some circumstances. It is important that if someone has a VAD request in place, that this CANNOT be overridden by the family. Otherwise it becomes too political and the clinicians are then held responsible for whichever decision they make in the end” *(Nurse).

 Violence from family towards staff was also a consideration.

*“Emotions running high from family members if they don’t agree or understand the patient’s request - we often see clashes from patient’s family members so I imagine a few code greys will occur” *(Nurse).

 Patients who requested VAD but were found to be ineligible could potentially be angry with staff and cause conflict.

*“It will be challenging for patients requesting it but who are not eligible, and it will be challenging to nurse these patients” *(Nurse).

 Some of the respondents were concerned that the community would have a negative perception of the hospital as a place which kills patients rather than cures them. There was some anxiety about potential picketing and protests outside the hospital.

*“Possibility of religious picketers outside of work (like for example the ones outside abortion clinics)” *(Nurse).

*“Misperception of being treated like an angel of death” *(Nurse).

###  Polarised Views About Voluntary Assisted Dying 

 There were some very strong views about VAD expressed in the survey results, ranging from whole-hearted rejection of VAD to total support. Many objected or were conflicted on the basis of religion.

 “[Challenge of] *reconciling my own religious beliefs, and ensuring I seek support when required” *(Allied Health).

 There were objections based on non-religious ethical concerns. Some respondents felt that VAD was contrary to the duty which clinicians owed their patients.

*“I am truly unwilling to participate in Voluntary Assisted Dying. As a registered nurse this act goes against everything I believe in. Every day, I am assisting patient to live, regain their health and leave the hospital. I also help patients during their last days, when illness becomes too much. I have a great love for this part of my job, it is a beautiful thing to make my patients comfortable and happy in their final hours… the act of assisting them in suicide rocks me to my core” *(Nurse).

 The relationship of trust currently between clinicians, patients and families was compromised by VAD in the opinion of some respondents.

*“It will also be even harder to provide proper palliative care now because there will be even less trust from patients and families, delaying proper palliative care further as conversations drag on.…This is so far out of bounds I don’t even know where to start…” *(Nurse).

*“My greatest reservation is because of my fear of the danger for our society and our civilisation consequent on the legalising of killing people” *(Medical Specialist).

 Some medical staff felt that the Hippocratic Oath would be contravened with any involvement in VAD and therefore was ethically unacceptable.

*“The Hippocratic Oath states that ‘Neither will I administer a poison to anybody when asked to do so, nor will I suggest such a course.’ Medicine has advanced to an incredible extent since this was written thousands of years ago. Yet the ethical and moral challenges involved with being a medical practitioner remain very similar” *(Medical Specialist).

*“This crosses an ethical line that we as medical physicians should not cross and opens out society to unforeseen consequences in relation to the value of a human life. We have a responsibility to ease suffering and heal where possible, but facilitating suicide (which is what VAD is, if we are honest about our terminology) is unacceptable” *(Medical Specialist).

 In contrast, there were others who strongly supported the idea of VAD, and many compared it to the withdrawal of treatment which was already practiced in many areas of the hospital.

*“I have worked at *[name withheld]* where withdrawal of ventilation was performed. Comfortable with idea of choosing when to die for incurable illness” *(Junior Medical Officer).

 VAD was seen by many to be a merciful end to intractable suffering. Poignantly, some of those who expressed this opinion cited their own experiences of watching patients and loved ones suffer at the end of life.

*“I wholeheartedly support VAD, I have seen too many people suffer unnecessarily in the past with terminal illness” *(Nurse).

*“My mother would have qualified for VAD. Unfortunately, it was not available at the time. I will never forget the pain she suffered” *(Nurse).

 Some respondents considered this issue to be all about the right of the patient suffering with a terminal illness to make decisions about their own life, and that the opinions of the clinicians were less important.

*“I believe it’s important that we remember we don’t deliver care based on subjective matters or personal opinions or beliefs… I 100% support this legislation and it will be a bitter-sweet moment/s in my life to be able to help provide warmth and comfort to my patients, their families and friends in the final moments of my patient’s life” *(Nurse).

###  Emotional Burden

 Almost all of the respondents expressed concern that VAD would result in an emotional burden for the staff involved. Witnessing the anguish of others and emotional farewell scenes would be challenging for many staff.

*“Regardless of whether a patient is VAD or palliated the situation is always challenging*.

*Listening to family / patient can be confronting. Supporting less experienced staff and keeping your own emotions in check”* (Nurse).

 Some mentioned feeling a sense of guilt about the part they would be playing in the patient’s death and being very stressed at potentially witnessing the process.

*“Possibly a minor feeling of guilt - even though you have respected a dying person’s wishes” *(Nurse).

*“Not knowing how I would feel knowing someone is about to end their life” *(Nurse).

 Formal emotional support and specific education were suggested for staff caring for patients undertaking VAD in order to off-set the emotional toll of this work.

*“I would like healthcare workers who participate in VAD to have to undergone mandatory counselling and psychology evaluation as to their ability to participate in the process” *(Nurse).

###  Vulnerable Patients 

 There was concern that vulnerable patients should be protected, specifically those who felt compelled to take the VAD option in order to relieve their families of burden, rather than because of their own desire to participate. Patients could potentially be coerced to opt for VAD rather than palliative care because it was quicker and cheaper. There was the possibility of abuse of power by staff or families who could exert undue influence over patients.

*“I would hate to ever see an environment where people felt pressured to utilise VAD because they felt they were a burden to family, staff or the system” *(Nurse).

 Participants suggested that the legislation could be misused by unscrupulous practitioners or families despite the safeguards in place.

*“I have major concerns about potential abuse of this legislation, against which the weakest and most vulnerable in our society will be the least able to defend themselves…despite the best intentions of our legal restrictions” *(Medical Specialist).

 In the opinion of some, ensuring that appropriate palliative care was available to patients at the end of life negated the need for VAD.

*“As an oncologist, I have very seldom encountered a patient whose terminal symptoms could not be adequately palliated as long as adequate resources were provided towards this” *(Medical Specialist).

 Evaluating the presence and treatment of depression was considered pivotal before implementing VAD to make sure that the patient was not unduly influenced by a temporary psychological state.

*“Assess the presence of clinical depression and the extent to which this is influencing the decision to use VAD legislation. Need to treat any depression before being able to access the legislation” *(Allied Health – Psychologist).

*“I fear killing the patient too soon or for the wrong reason for example they are depressed rather than truly terminal” *(Medical Specialist).

###  Organisational Challenges

 Many of the respondents were unclear about the details of the VAD process, including the role of the clinician, the process for referral, the storage and handling of the drug, the staffing and logistics.

 One issue which was mentioned by many respondents was that of staffing. They were concerned that clinicians would be expected to integrate a VAD procedure into the normal routine of the ward whereas they felt that they would need additional staffing resources and support.

*“As a nurse, I will not be administering the medication, but I do not simply walk out of the room once it is given… these events are able to be planned, and therefore, staffing should be allocated appropriately to support the patient and family through the process” *(Nurse).

 It was not only nurses who felt that they would require a different staffing level when caring for patients undergoing VAD, other disciplines were also doubtful about their ability to cope within existing staffing levels.

*“I feel that there will be an enormous increase in workload due to the significant emotional and psychological impact of VAD for the patient and their family. I also anticipate higher levels of family conflict as a result of VAD, which as social workers we are called upon to diffuse” *(Allied Health – Social work).

*“For medical staff, please facilitate an extra staff member into the team to dilute their workload, and allow them to purposefully participate, rather than become overburdened and withdraw” *(Nurse).

 As an alternative to increasing ward staffing in order to cope with VAD there were many suggestions about a specialist VAD team who could be called on to manage these patients.

*“… it would be helpful to have a team of dedicated people in the hospital who deal with VAD…” *(Allied Health – Social Work).

 Participants noted that the hospital environment can be noisy and busy with a lack of privacy in many instances. It was considered an unsuitable place for a death, especially one which could be forecast.

*“I think that it may be best to have a specialised place to conduct these deaths, and it should not be on the ward… It may also provide a quieter environment for family to grieve, any time day or night - not restricted by ward visiting hours” *(Nurse).

 The role of the nurse in the preparation and administration of the drug was not widely understood and caused some anxiety.

*“Drawing up medications and the legality and the nurses’ role in the actual VAD process. If the nurse has to sign as a witness or anything regarding to legal issues surrounding the VAD process” *(Nurse).

*“What medication will be given in the VAD process? What will the nurse need to expect when the medications are given (will it be pleasant / distressing) for the patient?” *(Nurse).

 Others also wanted more information about the process, including the mechanism of the drug and the timeline.

*“…need to know what the VAD drug was and mechanism of action and what would be expected after patient had been administered or taken the drug” *(Nurse).

 There was some concern about the storage and management of the lethal drug on a general ward.

*“…managing the logistics of the lethal drug, how would it be maintained with the patient, with the pharmacist or the health practitioner. How will it be kept even in the hospital?*”

*“…is there potential for paradoxical reactions? Also, what if patient has last minute panic? Could it be reversed?”* (Allied Health – Pharmacist).

 Many respondents mentioned the need for education and on-going support for staff involved in VAD. Education would need to include strategies for managing patient and family distress and conflict, as well as protocols and guidelines for staff. The specific role of the doctor/nurse/pharmacist/allied health would need to be made very clear to staff before proceeding with VAD.

*“Clear guidelines with detailed responsibilities and expectations allocated to all healthcare practitioners involved in this process. Escalation process should also be very clear from the outset and detailed education sessions would be beneficial - particularly being accessible to staff ie, tailored to nursing staff or medical staff” *(Junior Medical Officer).

###  Decision-Making

 Participants were concerned that the decision to opt for VAD could be difficult both for patients and families, and for the clinicians involved. Supporting a patient’s wish to die included ensuring that there was no abuse or coercion from other parties, and that other forms of treatment and symptom management had been thoroughly explored before VAD was approved. This included treating depression and providing psychological support and palliative care alternatives.

*“I am worried that some patients will decline the palliative measures that we can offer, thinking that VAD is the only option that will relieve their suffering, when they may be able to gain significant benefits in QOL *[quality of life] *from a very short period of palliative treatment…” *(Allied Health – Radiologist).

 There was some unease about determining patient capacity to provide consent, including from staff whose job included this task. Participants identified this as a challenge, noting that assessment of capacity can sometimes have a degree of subjective interpretation and may be viewed differently by different clinicians.

*“…if their capacity is unclear or there are different opinions on capacity in the team” *(Allied Health – Speech Pathologist).

*“Decision-making capacity - in relation to a patient making a decision for VAD, but also related issues eg, making a will, and implications for workload/boundaries of role, legal liabilities and hospital’s position if neuropsych is sued by family members challenging wills etc*” (Allied Health – Neuropsychologist).

 There was an issue raised about patients with cognitive or communication impairments who may wish to opt for VAD. Assessment of capacity to make the decision could be difficult and, in some cases, disputed.

*“Advocacy and managing conflict around decision-making capacity for patients with communication or cognitive impairments” *(Allied Health – Neuropsychologist).

 Additionally, patient prognosis was identified as a grey area in many cases, particularly difficulty determining if a patient has less than 6 months to live.

*“Correctly identifying difficult concepts i.e. when a patient is specifically going to die” *(Junior Medical Officer).

 Overall, clinicians who responded to the survey had some powerful insights into this divisive issue. Regardless of their specific view on VAD, many clinicians advocated thorough screening and monitoring of patients, and increased education and support for staff.

## Discussion

 The aim of this study was to explore the opinions of clinical staff about the implementation of the new VAD legislation in one Victorian healthcare institution. Diverse views were expressed, including staff vehemently opposed to VAD, others passionate in their support of VAD and many nuanced views in-between. Overall, respondents agreed that excellent quality care at the end of life was an important priority, but the place that VAD occupies within this care differed fundamentally.

 Many respondents felt that assisting a person to end their life was morally and ethically wrong. For some participants, this was linked with strong religious beliefs. This finding is supported by previous research which found that increased religiosity was equated with strong views against VAD.^21-23^ Others who objected to VAD believed that it contravened the ethical principles of beneficence and non-maleficence, or the Hippocratic Oath.^24^ For some, belief in the sanctity of life and the role of physicians to preserve life took priority over any other factors. In contrast, some participants argued that allowing a competent patient to make decisions about their end-of-life care was supporting the individual’s autonomy (ie, right to make their own decisions^3^) and expediting their wishes.^25^

 The protection of vulnerable patients reaching the end of life and suffering from the effects of a life-limiting illness was concerning to many respondents, with the right of the patient to make decisions, free from any controlling interference of others frequently discussed,^26,27^ This concern that in some situations, others may unduly influence the person to take the VAD option because it is perceived as more convenient, cheaper, quicker or less burdensome has been expressed in other literature.^28-30^ A similar problem is encountered when assessing patient capacity to request withdrawal of life-sustaining treatment when the circumstances and potential sequelae are open to interpretation , for example when a patient requests a ‘Do not resuscitate’ order when capacity is questioned or when further treatment is considered futile.^31,32^ In the current literature on VAD there are warnings about the ‘slippery slope’ which predicts the abuse of vulnerable groups in society such as the disabled,^33^ however other research does not support this finding^34,35^ To protect patients, avenues should be provided for confidential discussions with appropriately trained staff to ensure patients are making an autonomous choice.

 Some participants were concerned about the capacity of a patient to make an informed decision in the late stage of illness, especially when ‘unbearable suffering’ was present. This aligns with research indicating that a desire to die could be influenced by extreme, poorly controlled pain or other difficult symptoms such as acute dyspnoea, or depression.^22^ Supporting patient dignity and maintaining respect for the patient’s wishes in the face of imminent death is fundamental^5^and every effort must be made to ensure suffering is minimised so that patients can more fully engage in autonomous decision-making.^36,37^ Participants in this study were also concerned that determining decision-making capacity in the context of VAD was very difficult and could potentially lead to litigation from family or others. Tests designed to assess cognitive capacity, specifically the ability to engage in autonomous decision-making, are not definitive.^37^ The gravity of the decision means that the patient requires greater decision-making capacity than for other less serious decisions-making the assessment of capacity a crucial and important step.^26^

 Despite the challenges, it is important that the legal right of patients to explore this option is supported. Professional standards dictate that access to lawful health services is the right of the individual, despite the personal opinions of staff who may be opposed to VAD. Patients must have their symptoms including depression optimally managed before choosing VAD to ensure that their decision-making is not clouded by treatable conditions. Patients could potentially request VAD fearing pain and dependency, thus clinicians need sophisticated communication skills to determine if hidden issues need to be addressed before VAD is progressed^25,38^ and patient assessment must be comprehensive.^26^ There are additional concerns that patients may decline palliative care if VAD is an option despite palliative care being able to alleviate distressing symptoms in many cases. There are complex reasons that individuals seek VAD, and many of those who consider it do not ultimately progress to VAD.^28^ Comprehensive palliative care may alleviate physical symptoms and provide psychosocial and spiritual care and must always be available to patients considering VAD.^13,28,39^

 In this study, themes of conflict and emotional burden emerged. It has been reported in previous research that the effect of witnessing VAD or caring for patients taking this option has had a profound effect on some clinicians, ranging from intense professional fulfillment and reward^8,9^ through the whole spectrum of emotions to moral distress^9,40^ and concern for the wider implications for society in general.^26^ Many clinicians in our study highlighted the need for psychological support for staff involved in order to alleviate anxiety and limit the emotional burden and sense of responsibility they carry. The support and backing of the team is an important factor in lessening the stress on individual clinicians.^41^ It is also important that a clear, but empathetic approach is taken by hospital leadership to the range of staff views about controversial but lawful health options such as VAD. Developing an institutional approach that balances the needs of staff and patients is crucial.

 VAD is in its infancy in Australia. The present study provides an opportunity to understand the concerns of clinical staff and provide information which can inform practice.

###  Study Limitations

 A large number of clinicians from various disciplines participated in the survey and provided a broad range of opinion, showing a great variation in attitude to VAD. The limitations include that the survey responses were from one 600-bed acute hospital in Melbourne and may not accurately reflect the viewpoint of clinicians at other facilities. It is possible that clinicians with strong views were more likely to participate in the survey than others who were more equivocal. Further, this data was collected in the period between the legislation passing in Parliament and the start date for VAD in Victoria. The data thus represents the views of clinicians at one particular time, during which practical information on VAD in Victoria was very limited.

## Conclusion

 The aim of this study was to explore the opinions of clinical staff on the implementation of the new VAD legislation in one Victorian healthcare institution participating in VAD.

 The survey results show a high degree of clinician anxiety about the introduction of VAD at this hospital. Many of the respondents were unaware of the details of the legislation and had a poor understanding of their potential role in VAD or the process for patients. However, this could be related to the timing of the survey before implementation guidance materials were available. Staff require substantial education ideally delivered by leaders within the hospitals to clarify misconceptions about VAD and address staff concerns. Such education should build on exploration of VAD in tertiary health curricula, including exploration of the challenges of practising in a context of diverse views.

 In comparing this new Australian research with previous studies, it was clear that many of the challenges encountered here were previously found internationally. The respondents were united in their compassion for patients suffering at the end of life but had polarised opinions about VAD. Differences centred on religious and ethical objections conflicting with the rights of patients to self-determination and assisted death. There was considerable concern that these vulnerable patients must be protected from coercion, and that the capacity to make this permanent decision was firmly established before proceeding.

 Given the ongoing controversial nature of VAD, tolerance and respect for alternative viewpoints must be advocated in order to deliver the care that patients have the right to request.

## Acknowledgments

 We gratefully acknowledge input into the survey design by Dr. Bridget Pratt, A/Prof. Karen Detering, Dr. Marcus Sellars, Dr. Barbara Hayes, Mr. Mark Tacey, and A/Prof Anastasia Hutchinson.

## Ethical issues

 The study was approved by Austin Health Human Research Ethics Committee 45754/Austin 2018, and Alfred Health Human Research Ethics Committee 685/18.

## Competing interests

 DK is a member of the Victorian Voluntary Assisted Dying Review Board. This work was undertaken in her capacity as a palliative care and clinical ethics researcher, and not as a member of the Board. Any views expressed in this paper are DK’s personal views and are not to be attributed to the Review Board.

## Authors’ contributions

 Project design: TB, MG, RMcD, KO, LOD, DK. Survey design: RMcD, DK; Data analysis: RD, TB, LOD, RMcD; Manuscript preparation: RD; Manuscript review and editing: RD, TB, RMcD, KO, MG, LOD.

## Authors’ affiliations

^1 ^School of Nursing and Midwifery, Deakin University, Geelong, VIC, Australia. ^2^Centre for Quality and Patient Safety Research (QPS), Alfred Health Partnership, Melbourne, VIC, Australia. ^3^Melbourne School of Population and Global Health, University of Melbourne, Melbourne, VIC, Australia. ^4^Alfred Health Partnership, Melbourne, VIC, Australia. ^5^Austin Health, Melbourne, VIC, Australia. ^6^Department of Palliative Care, Austin Health, Melbourne, VIC, Australia. ^7^School of Nursing and Midwifery, Faculty of Health, Deakin University, Geelong, VIC, Australia.
